# NRP2^+^ human mesenchymal stem cells have stemness-associated properties

**DOI:** 10.1186/s41232-025-00376-3

**Published:** 2025-04-28

**Authors:** Kotaro Tanaka, Rintaro Yoshikawa, Satoru Miyagi, Takashi Suyama, Hiromi Miyauchi, Yuko Kato, Kenichi Miyamoto, Yumi Matsuzaki

**Affiliations:** 1 Department of Life Science, Faculty of Medicine, Shimane University, 89 - 1 Enya, Izumo, Shimane 693 - 8501 Japan; 2 Department of Plastic Surgery, Fukuoka Tokushukai Hospital, 4 - 5, Sugukita, Kasuga, Fukuoka Japan; 3 Department of Biochemistry, Faculty of Medicine, Shimane University, 89 - 1 Enya, Izumo, Shimane 693 - 8501 Japan; 4 PuREC Co., Ltd, 89 - 1 Enya, Izumo, Shimane 693 - 0021 Japan

**Keywords:** Mesenchymal stem cells, Neuropilin- 2, Stemness, Regenerative medicine, Bone marrow, Flow cytometry

## Abstract

**Background:**

The clinical application of mesenchymal stem cells (MSCs) has garnered attention due to their remarkable capacity to differentiate into adipocytes, chondrocytes, and osteoblasts. However, the quality of MSC culture varies from batch to batch, which poses challenges in ensuring consistent cellular quality across batches. Consequently, it becomes imperative to identify specific markers that can distinguish superior and slightly inferior MSCs.

**Methods:**

Human bone marrow-derived MSC clones were isolated and subjected to flow cytometry analysis to assess the expression of NRP2, VEGFR, and plexinA1. The osteogenic and adipogenic differentiation potentials were evaluated using Alizarin Red S and Oil Red O staining, respectively. Furthermore, the migration capacity was assessed through the scratch healing assay.

**Results:**

Nine out of twenty MSC clones significantly expressed NRP2. NRP2-expressing MSC clones (NRP2^+^ MSCs) retained superior proliferation and differentiation capacities, along with increased migratory capacity compared to non-expressing MSC clones (NRP2^−^ MSCs). In addition, the activation of VEGF-C/NRP2 signaling augmented the potential of MSCs in cell proliferation and differentiation.

**Conclusion:**

In contrast to NRP2^−^ MSCs, NRP2^+^ MSCs exhibited superior proliferation, differentiation abilities, and migration capacity. Moreover, the stimulation of VEGF-C/NRP2 signaling further enhanced the proliferation and differentiation rates, indicating a role of NRP2 in the maintenance of MSC stemness. Hence, NRP2 holds potential as a cell surface marker for identifying beneficial MSCs for regenerative medicine.

## Background

Mesenchymal stem cells (MSCs) have been attracting increasing attention due to their differentiation capacity into bone [1] and cartilage tissue [2] and their immunomodulatory function [3, 4] in cell therapy. Since the discovery, researchers have isolated MSCs from the bone marrow, adipose tissue, placenta, and dental pulp, based on adherence to plastic flasks, and harvested the adherent cells with a spindle-shaped morphology that emerge after 2–3 weeks of culture as MSCs. However, this conventional protocol is unable to establish the MSC culture, which is free from contamination of fibroblasts, granulocytes, and hematopoietic cells; these contaminants negatively affect transplantation outcomes [5]. Moreover, prolonged MSCs cultivation induces replicative senescence and compromises clinical outcomes. Senescent MSCs cause pulmonary embolism after intravenous transplantation because they fail to pass through the capillary vessels in the lung. Our previous study established a protocol to prospectively isolate MSC clones from the human bone marrow by flow cytometry using antibodies against low-affinity nerve growth factor receptor (LNGFR) and THY- 1. We demonstrated that the LNGFR^+^THY- 1^+^ fraction is enriched in colony-forming unit fibroblast (CFU-F) activity and a part of LNGFR^+^THY- 1^+^ cells clonally grow in a well of the 96-well plate even though the cell proliferation rate differs from clone to clone. The clones that grew to subconfluent in two weeks are called REC, Rapidly Expanding Clone. MEC, Moderately Expanding Clone, grew slower than REC, and SEC, Slowly Expanding Clone, merely expanded [6]. RECs also retain higher differentiation potential than MECs or SECs. Even in RECs, some clones cease proliferation at relatively early passages and undergo replicative senescence, whereas others maintain their differentiation and proliferative potential for a long-term. Consequently, cell surface markers that distinguish superior clones from inferior clones enable us to establish beneficial RECs for regenerative medicine. The International Society for Cellular Therapy defined the positive (CD105, CD73, and CD90) and negative markers (CD45, CD34, CD14 or CD11b, CD79α or CD19, and HLA-DR) for human MSCs [7]. However, these markers are minimal criteria of MSCs and do not necessarily guarantee the quality of MSCs.

NRP2 is a co-receptor for vascular endothelial growth factor receptor (VEGFR) and plexin that contributes to the arterial or venous identity of vessels in concert with NRP1 and sympathetic axonal responses to Semaphorin IV [8–11]. *NRP2* was also reported to be one of the genes expressed in human MSCs [12]; however, its role in MSCs remains unclear. Here, we demonstrated that the RECs with robust proliferation, differentiation, and migration potential express the NRP2. In addition, stimulation of VEGF-C/NRP2 signaling augments the proliferation and differentiation capacities. These results clearly show that NRP2 is an ideal marker for beneficial MSCs.

## Methods

### Isolation of bone marrow-derived MSCs and cell culture

Frozen human bone marrow mononuclear cells (BM-MNC) were purchased from Lonza, thawed at 37 °C and incubated in Hank’s Balanced Salt Solution (HBSS, Fuji Film Wako Pure Chemicals, Japan) containing 10 units/µL DNase I (Sigma-Aldrich) at room temperature for 10 min. The cell suspension was centrifuged at 200 × g for 5 min and the supernatant was discarded. The cells were resuspended with HBSS supplemented with 2% fetal bovine serum (FBS) and then stained with anti-LNGFR-APC (Miltenyi Biotec, Germany) and anti-THY- 1-PE (BioLegend, CA, USA). LNGFR^+^ THY- 1^+^ cells were sorted as single cells on each well of 96-well plates using JSAN JR (Bay Bioscience, Japan). Sorted cells were cultured in a growth medium (Dulbecco's Modified Eagle Medium [DMEM] supplemented with 20% FBS, 0.01 mol/L HEPES, 100 units/mL penicillin, 100 µg/mL streptomycin, and 20 ng/mL basic fibroblast growth factor [FGF]). The definition of MSC subtypes, REC, MEC, and SEC was described previously [6].

### Induction of osteogenic and adipogenic differentiation

MSCs were seeded on 12-well plates at a density of 1 × 10^5^ cells/well, and precultured in a growth medium until they became confluent. To induce osteogenic differentiation, the medium was replaced with an osteogenic induction medium (DMEM supplemented with 20% FBS, 0.01 mol/L HEPES, 100 units/mL penicillin, 100 µg/mL streptomycin, 10 mM β-glycerophosphate, 50 µM L-ascorbic acid, 0.1 µM dexamethasone, and 0.02% gentamicin). MSCs were cultured in the medium for 10 days, with the medium replaced every 3–4 days. To induce adipogenic differentiation, MSCs were cultured in an adipogenic induction medium (DMEM supplemented with 20% FBS, 0.01 mol/L HEPES, 100 units/mL penicillin, 100 µg/mL streptomycin, 0.5 mM isobutylmethylxanthine, 0.2 mM indomethacin, and 1 µM dexamethasone) for 10 days, with the medium replaced every 3–4 days. Osteogenic-induced cells and adipogenic-induced cells were stained with Alizarin Red S (Waldeck GmbH & Co KG, Division Chroma, Germany) and Oil Red O (MUTO PURE CHEMICALS, Japan), respectively. Briefly, osteogenic-induced cells were washed with phosphate-buffered saline (PBS), fixed with 4% paraformaldehyde (PFA) for 10 min, washed again with PBS three times, and then stained with Alizarin Red S for 5 min. Adipogenic-induced cells were washed with PBS and fixed with 4% PFA for 10 min. They were washed again with PBS three times, washed with 60% isopropyl alcohol for 30 s, and stained with Oil Red O for 30 min. Then cells were washed with 60% isopropyl alcohol for 30 s and washed with PBS three times. The stained cells were observed using BZ-X710 fluorescence microscope (KEYENCE, Japan). The differentiation capacity of MSCs was evaluated by measuring the ratio of the stained area using ImageJ software (National Institutes of Health).

### Flow cytometry

Cultured cells were detached from culture dishes using 0.05% trypsin- 0.53 mmol/L EDTA･4 Na solution, resuspended in 100 µL of HBSS containing 2% FBS for 1–3 × 10^5^ cells, and then stained on ice for 30 min with the following monoclonal antibodies and dilutions: anti-human/mouse NRP2-APC (1:10), anti-human VEGFR2-APC (1:20), and anti-human VEGFR3-APC (1:20). The cells were also treated with anti-human plexin A1 (1:100) and anti-mouse IgG2b-FITC (1:100) for primary and secondary antibodies, respectively. Anti-human/mouse NRP2-APC and anti-human PlexinA1 were purchased from R&D Systems (MN, USA). Anti-human VEGFR2-APC, anti-human VEGFR3-APC, and anti-mouse IgG2b-FITC were purchased from BioLegend. A flow cytometric analysis was performed on CytoFLEX (Beckman Coulter, CA, USA), and mean fluorescent intensity (MFI) was analyzed with FlowJo software (TreeStar, Inc., CA, USA).

### Scratch healing assay

The scratch healing assay was performed as described by Liang CC et al. previously [13]. After cells were seeded on 24-well plates and grown to confluence, one straight scratch wound was made in the monolayer using the CELL Scratcher (IWAKI, Japan). Then, the cells were washed using PBS to remove debris, and fresh DMEM (serum-free) was added. Representative images of the scratched areas were photographed at × 40 magnification under a light microscope to estimate the relative number of migrating cells. The migration area was defined as the area at which the cell-free area after 35–42 h of incubation was excluded from the total region scratched. The ratio of the migration area to the total scratched region was calculated.

### Quantitative reverse transcriptase polymerase chain reaction (q-PCR) analysis

Total RNA was purified with the RNeasy Mini Kit (Qiagen, Germany), and first-strand cDNA was synthesized with PrimeScript II Reverse Transcriptase (TaKaRa Bio, Japan). q-PCR was performed on the ABI 7500 Fast Real-time PCR System (Applied Biosystems, MA, USA) using the Fast SYBR Green Master Mix (Applied Biosystems, MA, USA). *NRP2* expression was analyzed using the ΔΔC_T_ method. The primer sequences were as follows: *NRP2*, 5’-CAGAATGGCTACTATGTCAAATCC- 3’ (forward) and 5’-CGTTGTTGGCTTGAAATACCT- 3’ (reverse); *Hprt*, 5’-TGACCTTGATTTATTTTGCATACC- 3’ (forward) and 5’-CGAGCAAGACGTTCAGTCCT- 3’ (reverse).

### Short-term cell proliferation assay

Five hundred cells were seeded on each well of a 96-well plate and cultured with or without 10 ng/mL of VEGF-C (BioLegend, CA, USA) for 7 days. Cell proliferation was measured using Cell Counting kit- 8 (CCK- 8, Dojindo, Japan) according to the manufacturer’s instructions. After the working solution of CCK- 8 was added to each well, cells were incubated in a humidified incubator with 5% CO_2_ at 37 °C for 2 h. The absorbance was measured at 450 nm using GloMax Discover microplate reader (Promega, USA).

### Long-term cell proliferation assay

As the start point of the experiment, MSCs at passage 4 were seeded on 10-cm dishes at a density of 2 × 10^5^ cells/dish. These cells were cultured until they became confluent, treated with 0.05% trypsin- 0.53 mmol/L EDTA･4 Na solution, harvested, and then counted using hemocytometers. They were seeded again at a density of 2 × 10^5^ cells/dish. The counting and seeding of cells were repeated until passage 10.

### Microarray analysis

Transcriptome profiling was performed on three RECs and four MECs using a GeneChip Array System (Applied Biosystems, MA, USA). Briefly, total RNA was isolated using TRIZOL solution (Invitrogen, MA, USA), and 250 ng of the total RNA was amplified and labeled with biotin using the GeneChip 3'IVT Reagent Kit (Applied Biosystems, MA, USA). Ten micrograms of the labeled cRNA was hybridized using the Human Genome U133 Plus 2.0 Array. After hybridization and washing with the Hybridization, Wash, and Stain Kit (Applied Biosystems, MA, USA), images were obtained using Affymetrix GeneChip Command Console software. All procedures were conducted according to the manufacturer’s instructions. Global scaling, which sets the average signal intensity of all probes to a Target Signal of 500, was performed using Affymetrix Expression Console software. Normalized data was analyzed using limma and affy package from Bioconductor [14]. A heatmap was created using Prism software (GraphPad).

### Statistical analysis

Data are shown as the mean ± standard deviation (SD). The significance of differences was assessed by Student’s *t*-test, and *P* values of < 0.05 were considered significant.

## Results

### *NRP2* is strongly expressed in RECs

**Fig. 1:**
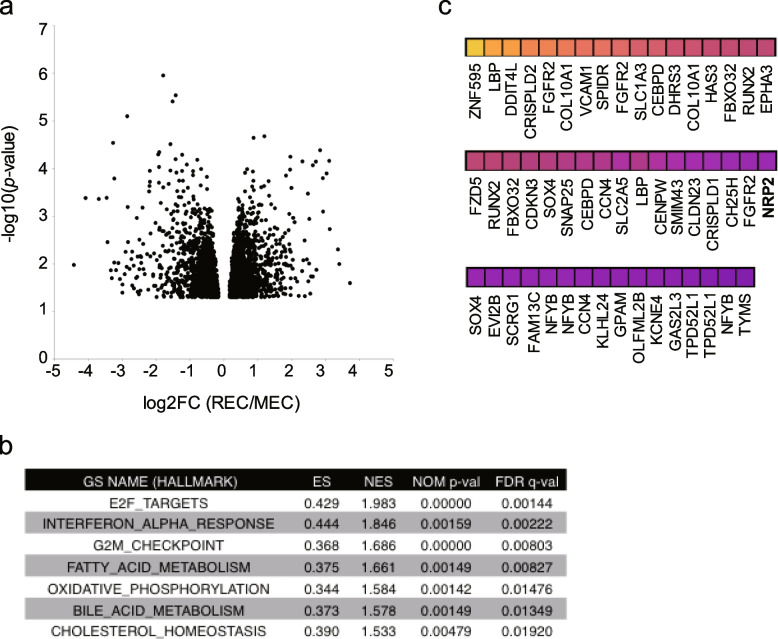
NRP2 is strongly expressed in RECs. **a** Volcano plot showing differences in the gene expression profile of RECs and MECs. Only probes with *p* value of less than 0.05 were plotted. **b** Gene sets enriched in RECs. Normalized enrichment scores (NES), nominal *p* values (NOM), and false discovery rates (FDR) are indicated. **c** The TOP50 of up-regulated genes in RECs are shown as a heatmap. MEC, moderately expanding clone; REC, rapidly expanding clones

To screen the genes that could serve as cell surface markers for MSCs with stem cell characteristics, we performed microarray analysis using three REC clones and four MEC clones. Statistical analysis identified approximately 2200 genes (3187 probes) upregulated in REC clones in comparison with MEC clones with a *p*-value of less than 0.05 (Fig. 1a). Gene set enrichment analysis (GSEA) revealed the positive enrichment of gene sets related to Cell cycle, Interferon response, Lipid metabolism, and Oxidative phosphorylation in REC (Fig. 1b). Among TOP50 genes (Fig. 1b), we focused on *Neuropilin 2* (*NRP2)* because the gene encodes a cell surface protein and its role in MSCs has yet to be reported (Fig. 1c).

### RECs include NRP2^+^ and NRP2^−^ clones

**Fig. 2:**
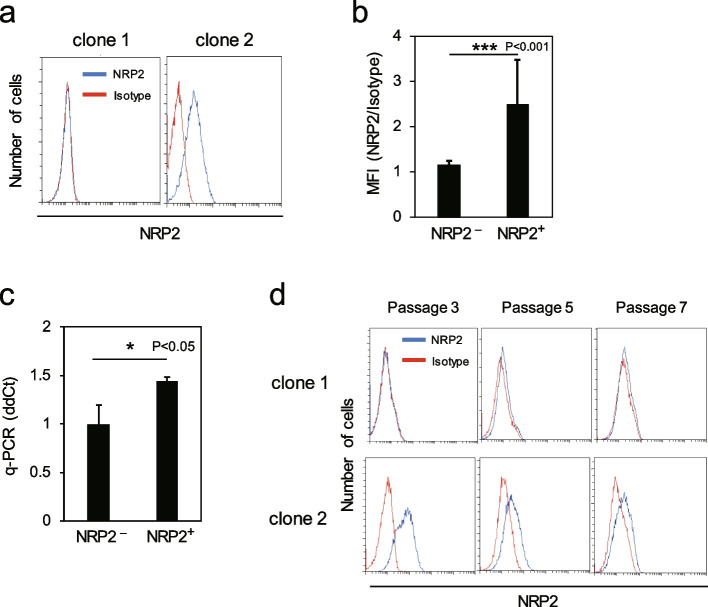
RECs include NRP2^+^ and NRP2^−^ clones. **a** Representative data of NRP2 expression levels in NRP2^−^ and NRP2^+^ clones (*n* = 11 or 9 per group). **b** MFI values of NRP2-APC on NRP2^–^ and NRP2^+^ clones. Values are represented as the mean ± standard deviation (SD) (*n* = 3). (c) q-PCR analysis of NRP2 expression in NRP2^−^ and NRP2^+^ clones. Values are represented as the mean ± SD (*n* = 3). (d) Expression levels of NRP2 in NRP2^−^ and NRP2^+^ clones at passages 3, 5, and 7 were analyzed by flow cytometry. *p < 0.05, ***p < 0.001. q-PCR, quantitative polymerase chain reaction; REC, rapidly expanding clones

The cell surface expression levels of NRP2 in RECs at passage 3 were analyzed by flow cytometry and 9 out of 20 MSC clones significantly expressed NRP2. Representative data are shown in Fig. 2a. Interestingly, *NRP2* expression levels differed in each REC, i.e., some exhibited slightly dim but single peak expression of *NRP2*, whereas others did not (Fig. 2a). A comparison of MFI in NRP2^+^ and NRP2^−^ RECs showed a significant difference between the two groups (Fig. 2b). A q-PCR analysis confirmed that the gene expression of *NRP2* was higher in NRP2^+^ RECs than in NRP2^−^ RECs (Fig. 2c). Then, changes in the expression levels of NRP2 were investigated among serial passages. The results revealed that the expressions were not detected in NRP2^−^ RECs throughout the culture period. On the contrary, the intensity of the expression level of NRP2 in NRP2^+^ RECs was relatively high in the early passage and gradually reduced as the passage number increased (Fig. 2d).

### NRP2^+^ RECs have a higher proliferation and differentiation capacity than NRP2^−^ RECs

**Fig. 3:**
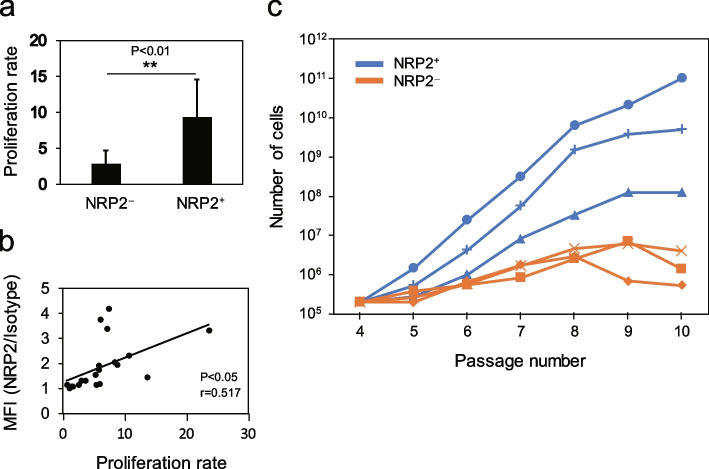
NRP2^+^ RECs have a higher proliferation capacity than NRP2^−^ RECs. **a** Proliferation rates of NRP2^−^ and NRP2^+^ RECs at passage 3 (*n* = 11 or 9 per group). A total of 1 × 10^5^ cells were seeded on 10-cm culture dishes, and the number of cells was counted after 5 days. **b** Correlation between the proliferation rate and expression level of NRP2. **c** Growth potential of representative NRP2^+^ and NRP2^−^ RECs from passages 4–10. REC, rapidly expanding clones

Owing to the variations in the properties of each REC, such as proliferation and differentiation potentials, we hypothesized that the expression level of *NRP2* may be related to such functions. Expectedly, the proliferation rate of NRP2^+^ RECs was significantly higher than that of NRP2^**−**^ RECs at passage 3 (Fig. 3a). The proliferation rate and NRP2 expression level were significantly correlated (Fig. 3b). In the long-term serial passage, NRP2^+^ RECs exhibited higher long-term growth potential than NRP2^**−**^ RECs (Fig. 3c). These results suggested that NRP2^+^ RECs have a higher proliferation capacity than NRP2^**−**^ RECs in short- and long-term in vitro cultures.

**Fig. 4:**
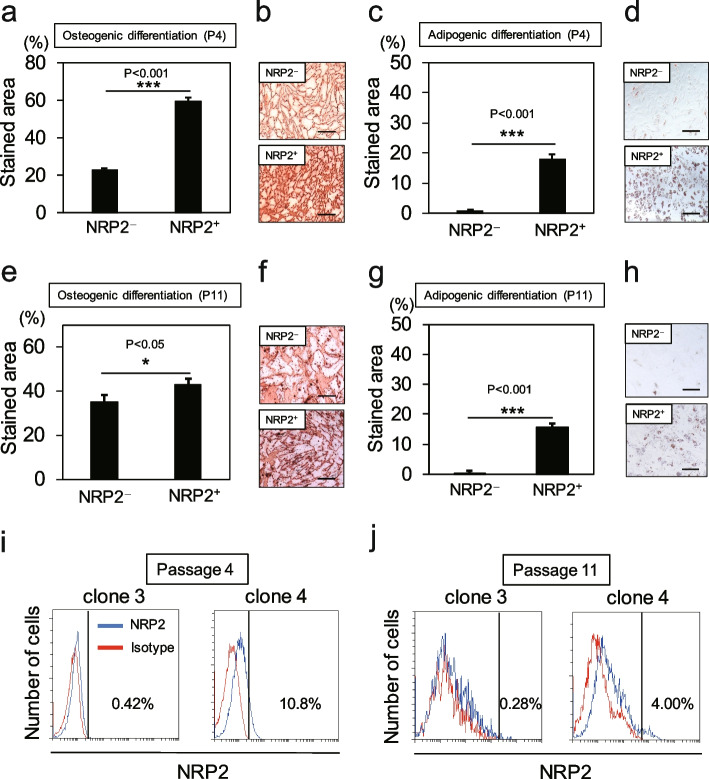
NRP2^+^ RECs have a higher differentiation capacity than NRP2^−^ RECs. (**a**, **b**, **e**, **f**) Osteogenic potential of each REC at passage 4 (**a**, **b**) and passage 11 (**e**, **f**). The percentage of the area stained with Alizarin Red S was calculated, and representative micrographs are shown. Values are represented as the mean ± standard deviation (SD) (*n* = 3). Scale bar = 300 µm. (**c**, **d**, **g**, **h**) Adipogenic potential of each REC at passage 4 (**c**, **d**) and passage 11 (**g**, **h**). The percentage of the area stained with Oil Red O was calculated, and representative micrographs are shown. Values are represented as the mean ± SD (*n* = 3). Scale bar = 300 µm. (**i**, **j**) NRP2 expression levels in NRP2^+^ and NRP2^−^ RECs at passages 4 and 11 analyzed by flow cytometry. *p < 0.05, ***p < 0.001. REC, rapidly expanding clones

Then, the ability of NRP2^+^ and NRP2^**−**^ RECs at passages 4 and 11 to differentiate into osteocytes and adipocytes was investigated. At passage 4, the differentiation potential of NRP2^+^ RECs was significantly higher than that of NRP2^**−**^ RECs in both osteogenesis and adipogenesis (Fig. 4a–d). The differentiation potential at passage 11 was similar to that at passage 4 (Fig. 4e–h). We also confirmed that the expression levels of NRP2 were higher in NRP^+^ RECs than in NRP2^**−**^ RECs at passages 4 and 11 (Fig. 4i, j). Therefore, compared with NRP2^−^ RECs, NRP2^+^ RECs at early and late passages exhibited higher differentiation ability to osteocytes and adipocytes in vitro. These results suggest that the expression levels of NRP2 are highly correlated with the proliferation and differentiation potency of RECs.

### Proliferation and differentiation capacities of NRP2high and NRP2low populations in one NRP2^+^ REC do not markedly differ

**Fig. 5:**
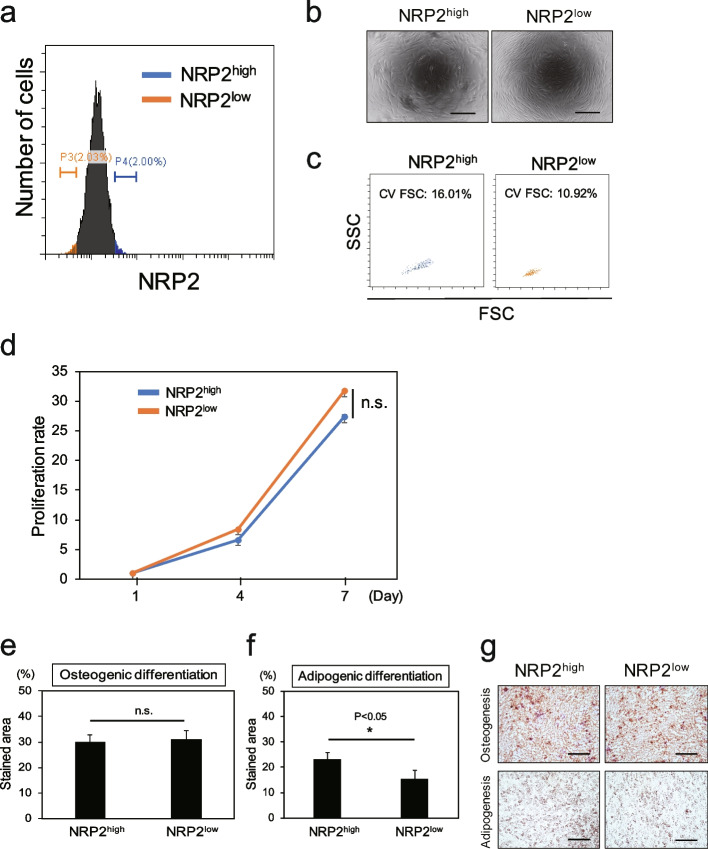
Proliferation and differentiation capacities of NRP2^high^ and NRP2^low^ populations in one NRP2^+^ REC did not markedly differ. **a** NRP2^high^ and NRP2^low^ populations in one NRP2^+^ REC were defined by flow cytometry. **b** Phase-contrast micrographs of NRP2^high^ and NRP2^low^ populations. Scale bar = 300 µm. **c** The cell size of each population was analyzed by flow cytometry. **d** The proliferation rates of each population were measured for 7 days. Data are represented as the means ± standard deviation (SD) of triplicate experiments. (*n* = 1) **e** Osteogenic potential of each population. The percentage of the area stained with Alizarin Red S was calculated. Data are represented as the means ± SD of triplicate experiments. **f** Adipogenic potential of each population. The percentage of the area stained with Oil Red O was calculated. Data are represented as the means ± SD of triplicate experiments. **g** Representative micrographs of osteogenic- and adipogenic-induced cells derived from NRP2^high^ and NRP2^low^ populations. Scale bar = 300 µm. n.s., not significant. *p < 0.05. REC, rapidly expanding clones

To investigate the correlation between the proliferation and differentiation capacities and NRP2 expression level in one NRP2^+^ REC, NRP2^high^ and NRP2^low^ populations were sorted based on the gating of top ~ 2% and bottom ~ 2% of NRP2-expressing cells in an NRP2^+^ REC (Fig. 5a). In our previous observation, MECs and SECs, which were composed of larger cells than RECs, had a lower proliferation and differentiation potential than RECs [6]. Therefore, we compared the cellular sizes between NRP2^high^ and NRP2^low^ cells. As shown in Figure 5b and c, NRP2^high^ cells tended to be larger than NRP2^low^ cells. However, the proliferation rates of NRP2^high^ and NRP2^low^ cells were not significantly different, even though NRP2^low^ cells proliferated slightly faster than NRP2^high^ cells (Fig. 5d). Moreover, the differentiation potential of these two groups into osteocytes and adipocytes was compared, and no significant differences in osteogenic differentiation were found. However, the adipogenic differentiation potential of NRP2^high^ cells was higher than that of NRP2^low^ cells (Fig. 5e–g).

### NRP2^+^ RECs have a higher migration capacity than NRP2^−^ RECs

**Fig. 6:**
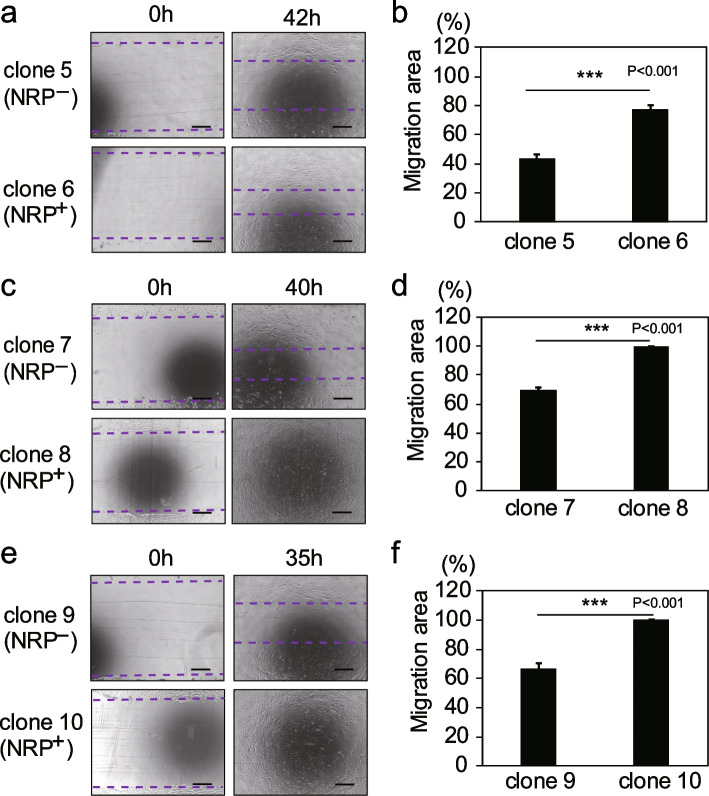
NRP2^+^ RECs have a higher migratory capacity than NRP2^−^ RECs. **a** Representative micrographs of scratched areas 0 and 42 h after the scratch wound were made. Clone 5 was the NRP2^−^ REC, and clone 6 was the NRP2^+^ REC. Scale bar = 500 µm. The dotted line represents the migration front. **b** The ratio of the migration area to the total scratched region was calculated. Data are represented as the means ± standard deviation (SD) of triplicate experiments. **c**–**f** Representative micrographs of scratched areas 0 and 35–40 h after the scratch wound was made (**c**, **e**) and the ratio of the migration area to the total scratched region (**d**, **f**). Clones 7 and 9 were NRP2^−^ RECs, and clones 8 and 10 were NRP2.^+^ RECs. Scale bar = 500 µm. The dotted line represents the migration front. Data are represented as the means ± SD of triplicate experiments. ***p < 0.001

Then, the motility of NRP2^+^ and NRP2^−^ RECs was examined. Figure 6a shows that NRP2^+^ REC (clone 6) migrated farther than NRP2^−^ REC (clone 5) 42 h after scratching (Fig. 6a, b). In addition, similar results were obtained using different clones (NRP2^+^: clones 8 and 10, NRP2^−^: clones 7 and 9) (Fig. 6c–f). These results indicate that NRP2^+^ RECs have a higher migration capacity than NRP2^−^ RECs.

### Effects of VEGF-C/NRP2 signaling on MSCs

**Fig. 7:**
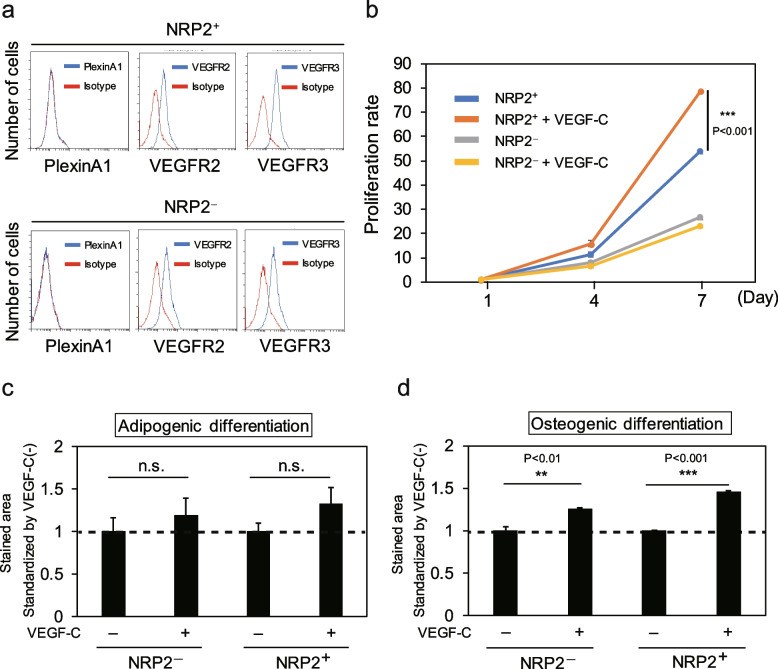
Effects of VEGF-C/NRP2 signaling on MSCs. **a** The expression levels of plexinA1, VEGFR2, and VEGFR3 on NRP2^−^ and NRP2^+^ RECs were analyzed by flow cytometry. **b** Representative proliferation rates of NRP2^+^ and NRP2^−^ RECs cultured with or without VEGF-C were measured for 7 days (*n* = 3 per group). Data are represented as the means ± standard deviation (SD) of triplicate experiments. **c** Representative adipogenic potential of each REC cultured with or without VEGF-C (*n* = 3 per group). After the percentage of the area stained with Oil Red O was calculated, its value was standardized by the value of each REC cultured without VEGF-C. Data are represented as the means ± SD of triplicate experiments. **d** Representative osteogenic potential of each REC cultured with or without VEGF-C (*n* = 3 per group). After the percentage of the area stained with Alizarin Red S was calculated, its value was standardized by the value of each REC cultured without VEGF-C. Data are represented as the means ± SD of triplicate experiments. n.s., not significant. **p < 0.01, ***p < 0.001. REC, rapidly expanding clones

Since *NRP2* was reported to constitute a co-receptor with plexinA1, VEGFR2, and VEGFR3, their cell surface expression in MSCs was analyzed. As shown in Fig. 7a, neither NRP2^+^ nor NRP2^−^ RECs expressed plexinA1 but expressed VEGFR2 and VEGFR3. Then, whether VEGF-C/NRP2 signaling affected the proliferation rate of MSCs was investigated. The growth rate of cells was assessed in a medium with or without VEGF-C supplementation for 7 days. The results showed that NRP2^+^ RECs in a medium supplemented with VEGF-C proliferated significantly faster than those in a medium without VEGF-C (Fig. 7b). In contrast to NRP2^+^ RECs, the growth rate of NRP2^−^ RECs did not enhance by VEGF-C.

VEGF promotes the osteogenic differentiation of MSCs [11, 15]. Therefore, whether stimulation with VEGF-C affected differentiation into osteoblasts and adipocytes was investigated. Although no significant difference in adipogenic differentiation was found between cells with and without VEGF-C (Fig. 7c), VEGF-C significantly enhanced the osteogenic differentiation of NRP2^+^ and NRP2^−^ RECs (Fig. 7d). As with the proliferation rate, the effect of VEGF-C on NRP2^+^ RECs was greater than that on NRP2^−^ RECs for osteogenic differentiation. These results suggest that VEGF-C/NRP2 signaling in MSCs significantly affects proliferation and differentiation into osteoblasts.

## Discussion

MSCs can be expanded in culture to the amount that meets the clinical requirements. However, long-term serial passaging accompanies the replicative senescence, which hampers cell proliferation and differentiation capacity. The capacity of MSCs to expand differs from batch to batch. Therefore, screening the beneficial MSCs in MSC-based regenerative medicine before clinical usage is crucial. This study demonstrated that approximately 45% of REC clones express NRP2 (NRP2^+^ RECs), and the expression in NRP2^+^ RECs ceases upon serial passaging. NRP2^+^ RECs robustly proliferate and differentiate into adipocytes and osteoblasts, demonstrating that NRP2^+^ RECs retain stem cell properties. Contrary, NRP2^−^ RECs show poor proliferation and differentiation capacity. These results clearly show that NRP2 expression distinguishes beneficial MSCs from adverse MSCs. CD90/THY- 1 is a well-recognized MSC marker. Given that CD90 expression is one of the minimal criteria of MSCs, it is supposed to be expressed virtually in all MSCs independent of their proliferation and differentiation capacity. Therefore, NRP2 is a superior cell surface marker to CD90 for screening of beneficial MSCs.

The neuropilin family has two members, *NRP1* and *NRP2*. Previous studies have reported that NRP1 is a marker of human bone marrow mesenchymal stromal cell-derived extracellular vesicles and regulates osteogenesis [16, 17]. Human MSCs also express NRP2 [12]. *Nrp2* deletion reduces bone mass in mice, indicating its role in osteogenesis [18]. VEGF-C, one of the ligands of NRP2, promotes the osteogenic differentiation of MSCs [15]. In this study, we demonstrated that VEGF significantly enhances the proliferation and osteogenic differentiation of NRP2^+^ MSCs, but not NRP2^−^ MSCs, showing that VEGF fulfills its function through NRP2 in MSCs. In addition, VEGF tends to enhance adipogenic differentiation. Therefore, it is worth speculating that VEGF/NRP2 signaling plays a role in maintaining the self-renewal capacity of MSCs, rather than simply promoting cell proliferation. We also note that this is the first to report the role of *NRP2* in MSCs. However, it will be necessary to conduct the gain- and loss-of-function analysis of the NRP2 gene to confirm the functional role of *NRP2* in MSCs. Moreover, Elaimy et al. reported that transcriptional coactivator with PDZ-binding motif (TAZ) is the downstream effector of VEGF/NRP2 signaling in breast cancer cells [19]. Therefore, TAZ is also possibly involved in the proliferation and differentiation of MSCs.

In the present study, NRP2^+^ RECs showed higher proliferation, differentiation, and migration potentials than NRP2^−^ RECs, and VEGF-C notably enhanced the proliferation and differentiation rates of NRP2^+^ RECs. We observed that all LNGFR^+^THY- 1^+^ cells expressed NRP2 before culture (data not shown). Therefore, it is plausible to conclude that only MSCs with stemness-associated properties can maintain NRP2 expression for a prolonged period after isolation while all MSCs express NRP2 when they reside in the bone marrow. Although the exact mechanism underlying the maintenance of NRP2 expression remains unclear, it is likely influenced by differences in their epigenetic status and/or the circumstances in which they reside in the bone marrow.

## Conclusions

This study demonstrated that NRP2^+^ RECs had robust proliferation, differentiation, and migration potentials. Establishing a technique to screen MSCs with stemness-associated properties at a higher rate in the initial stage of culture will significantly contribute to the advances in regenerative medicine. Therefore, NRP2 is a marker of MSCs with stemness-associated properties.

## Data Availability

All data generated or analyzed during this study are available within the manuscript or from the corresponding author upon reasonable request.

## Abbreviation

MEC: Moderately expanding clone
MSCs: Mesenchymal stem cells
NRP2: Neuropilin-2
REC: Rapidly expanding clone
SEC: Slowly expanding clone
TAZ: Transcriptional coactivator with PDZ-binding motif
VEGF: Vascular endothelial growth factor
VEGFR: Vascular endothelial growth factor receptor
